# Pharmacy-based predictors of non-adherence, non-persistence and reinitiation of antihypertensive drugs among patients on oral diabetes drugs in the Netherlands

**DOI:** 10.1371/journal.pone.0225390

**Published:** 2019-11-15

**Authors:** Sofa D. Alfian, Petra Denig, André Coelho, Eelko Hak

**Affiliations:** 1 Unit Pharmaco-Therapy, -Epidemiology & -Economics, Groningen Research Institute of Pharmacy, University of Groningen, Groningen, the Netherlands; 2 Department of Pharmacology and Clinical Pharmacy, Faculty of Pharmacy, Universitas Padjadjaran, Sumedang, Indonesia; 3 Department of Clinical Pharmacy and Pharmacology, University of Groningen, University Medical Center Groningen, Groningen, the Netherlands; 4 Health & Technology Research Center (H&TRC), Escola Superior de Tecnologia da Saúde, Instituto Politécnico de Lisboa (ESTeSL), Lisbon, Portugal; Boston University, UNITED STATES

## Abstract

**Background:**

Adherence to antihypertensive drugs in patients with diabetes is important. To support adherence, attention should be paid to the dynamic process of implementation, persistence and reinitiation of these drugs. We assessed non-adherence, non-persistence and reinitiation patterns for antihypertensive drugs in patients on oral diabetes drugs and identified pharmacy-based predictors of these processes.

**Methods:**

We conducted a cohort study in patients on oral diabetes drugs who initiated antihypertensive drugs between 1995–2015, as registered in the IADB.nl pharmacy database. Non-adherence was defined as a medication possession ratio < 80% and non-persistence as a gap > 180 days. We defined reinitiation as the dispensing of an antihypertensive drug within one year following discontinuation. We provide descriptive statistics for different time periods and applied logistic and Cox regressions to assess associations with sociodemographic and drug-related factors.

**Results:**

Of 6,669 initiators, non-adherence rates in persistent patients decreased from 11.0% in the first year to 8.5% and 7.7% in the second and third years, respectively. Non-persistence rates decreased from 18.0% in the first year to 3.7% and 2.9% in the second and third years, respectively. Of the 1,201 patients who discontinued in the first year, 22.0% reinitiated treatment within one year. Non-adherence and non-persistence rates were lower in the more recent time period. Predictors of non-adherence were secondary prevention (OR: 1.45; 95% CI: 1.10–1.93) and diuretics as initial drug class (OR: 1.37; 95% CI: 1.08–1.74). Predictors of non-persistence were female gender (HR: 1.18; 95% CI: 1.05–1.32), older age (HR: 1.33; 95% CI: 1.08–1.63) and diuretics, beta-blocking agents or calcium channel blockers as initial drug class. Longer duration of persistence was a predictor of reinitiation.

**Conclusions:**

Adherence to antihypertensive drugs in patients on oral diabetes drugs has improved over time. The first year after initiation is the most crucial with regard to non-adherence and non-persistence, and the risk groups are different for both processes. Early non-persistence is a risk factor for not reinitiating treatment.

## Introduction

Hypertension is common in patients with diabetes and contributes significantly to an increased risk of cardiovascular disease (CVD).[1] In the Netherlands, the guideline for cardiovascular risk management (CVRM) recommends antihypertensive drug treatment for patients with type 2 diabetes and elevated blood pressure (SBP ≥ 140 mmHg).[2] Although antihypertensive drugs are effective, adherence to these drugs in patients with type 2 diabetes is known to be suboptimal.[3] The first year of therapy has been identified as the highest risk period for non-adherence to and non-persistence with antihypertensive drugs.[4,5] Early interventions to support optimal drug-taking behaviour is important since hypertension is an asymptomatic chronic condition that requires long-term treatment.[1] Therefore, guidelines emphasize checking patients’ adherence when antihypertensive drugs have insufficient effect.[2] Furthermore, as patient behaviour is modifiable, pharmacists and other healthcare workers require information to identify which patients are in need of close monitoring and early intervention, preferably from readily available data.

Previous cohort studies showed that antihypertensive drug use is a dynamic process.[6–9] Even if the patient persistently uses the drug, non-adherence to the drug may occur.[10] Conversely, non-persistent users may reinitiate treatment.[6–9] However, a major flaw of many studies is the lack of a clear distinction between non-adherence and non-persistence and the failure to address reinitiation in the same population. Moreover, the generalizability of the previous findings to the high-risk group of patients with type 2 diabetes–for which there has been much effort to improve the quality of care in the past decade–is uncertain.

To develop tailored intervention, information is needed on specific predictors of these separate processes of drug-taking behaviour, and this is available to pharmacists in their dispensing records. The primary objectives of this study were to identify pharmacy-based predictors of non-adherence and non-persistence in the first year after initiation of an antihypertensive drug among patients on oral diabetes drugs, and to identify pharmacy-based predictors of reinitiation of antihypertensive drugs within one year after discontinuation among these patients. The secondary objectives were to describe patterns of non-adherence, non-persistence and reinitiation of antihypertensive drugs as discrete and dynamic processes among these patients and to compare these patterns across different time periods.

## Methods

We followed the Recommendations for Evaluating Compliance and Persistence with Hypertension Therapy using Retrospective Data[11], and the Medication Adherence Reporting Guideline published by the European Society for Patient Adherence, Compliance, and Persistence (ESPACOMP).[12]

### Study design and data source

We designed an observational retrospective inception cohort study using data from the University of Groningen pharmacy database IADB.nl. The IADB.nl pharmacy database contains anonymized and coded drug-dispensing data for more than 20 years, collected from 60 community pharmacies in the Netherlands and covering a dynamic annual population of approximately 600,000 people.[13] In accordance with the Dutch Medical Research Involving Human Subjects Act[14], ethics committee approval was not required because research using anonymous records in the Netherlands does not warrant it.

### Study population

The study population consisted of patients with type 2 diabetes who initiated an antihypertensive drug in the study period anywhere between 1995 and 2015, and who were registered in the IADB.nl pharmacy database at least one year before and after the first prescription of the antihypertensive drug. Type 2 diabetes was defined by the dispensing of at least two prescriptions for a non-insulin blood-glucose lowering drug without or with concurrent prescriptions for insulin within one year before the index date for patients aged 40 years and older. Younger patients were excluded to avoid misclassification of a patient that did not have type 2 diabetes. The index date was defined as the first dispensing of a diuretic, a beta-blocking agent, a calcium channel blocker or an agent acting on the renin-angiotensin system, with no dispensing of any of these drugs in the preceding 365 days. Patients dispensed with three or more of these drug classes within seven days from the index date were excluded because it was unlikely that this referred to initiation of antihypertensive treatment. In Dutch outpatient care, it is not recommended to prescribe three or more drugs within seven days of initial treatment. Patients who initiated propranolol were excluded because this drug can be potentially prescribed in prophylactic treatment of migraine. Patients who initiated high-ceiling diuretics were excluded because this drug is often intended for short-term use.

### Outcome measures

**Fig 1:**
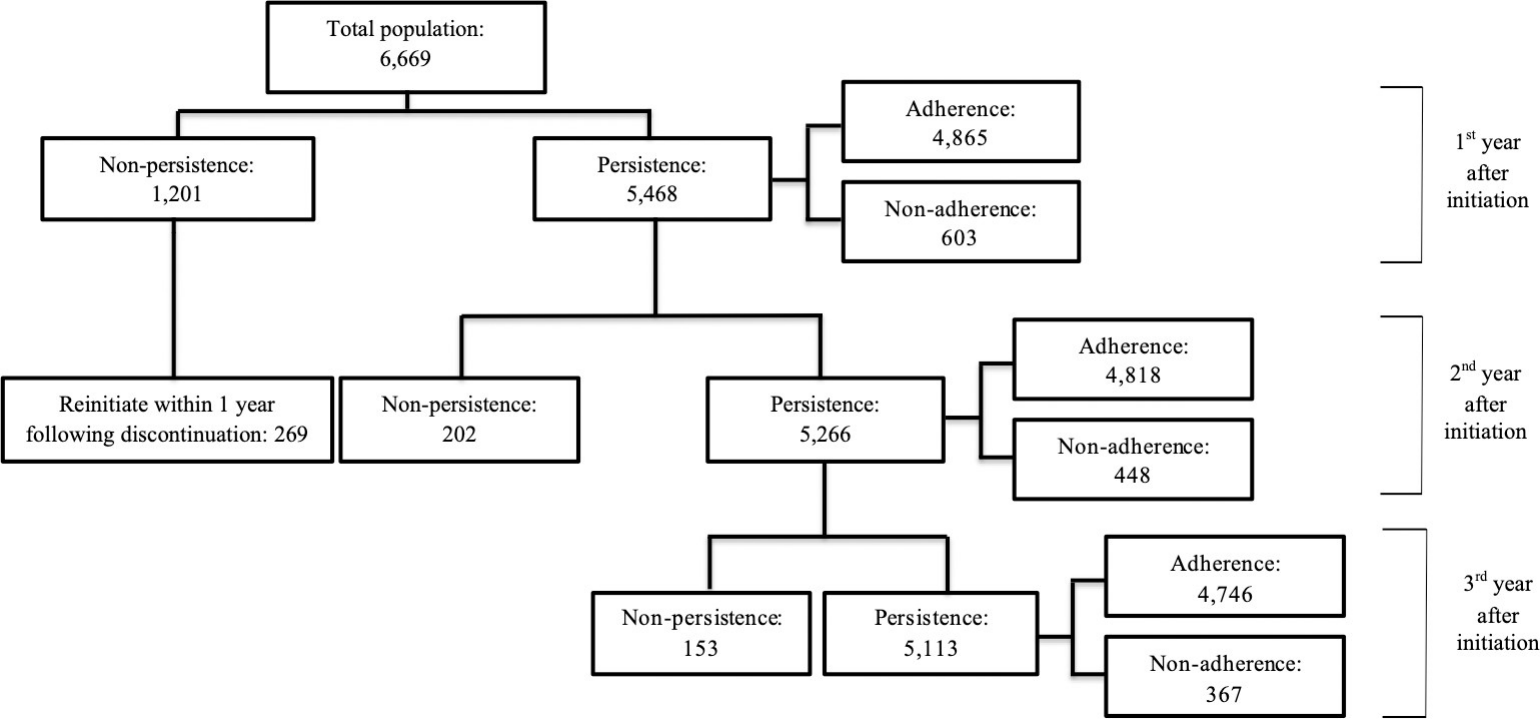
Flowchart of numbers of non-adherent and non-persistent patients and those who reinitiated antihypertensive drugs over three years.

Adherence and persistence rates were calculated for the first, second and third years after initiation of an antihypertensive drug. The one-year reinitiation rate was calculated among those who discontinued in the first year (S1 Fig). Non-adherence was calculated in persistent patients and defined as a medication possession ratio (MPR) < 80% for any antihypertensive drug. In other words, the MPR was calculated for patients without a gap > 180 days, and defined as the number of days’ supply divided by the number of days between the start of the first and the end of the last prescription in each year (i.e. prescription-based approach). This calculation was based on the taxonomy for describing and defining adherence to medications developed by Vrijens et al., who emphasize that non-adherence and non-persistence occur in different phases of the process.[10] MPR was capped at 1. Fixed-dose combinations were counted as one drug class in the MPR calculation. Patients were considered non-adherent if at least one antihypertensive drug had an MPR < 80%, that is, not adherent to any of their antihypertensives.[15] Patients were excluded from the MPR calculation in the years after they had become non-persistent (Fig 1). Drug switches were defined as the start of a new antihypertensive drug within < 180 days of the discontinuation (gap > 180 days) of another antihypertensive drug. In the case of a switch within the same drug class level (drugs with a similar mechanism of action), calculation of the MPR included the supply of both drugs, assuming that the individual drugs within the same drug class were interchangeable in terms of adherence calculation to multiple drugs. If a patient switched to a new drug within a drug class before the end of the last prescription of the previous drug, the overlapping days were shifted forward. We assumed that the new drug was taken after the previous drug had run out, since such within-class switches may be caused by reimbursement or supply issues. In the case of switches between drug class levels, the MPRs were calculated for each drug class separately as the number of days’ supply divided by the number of days between the start of the first prescription until the end of the last prescription.

Persistence was defined as continuously refilling a prescription for any antihypertensive drug without a gap > 180 days since the end of the last prescription[15], regardless of drug switches within or between drug classes or add-on drugs during follow-up. In the Netherlands, the average repeat prescription length for drugs used for chronic diseases is 90 days. A gap length of twice the prescription duration of 180 days was chosen to clearly distinguish patients who discontinued treatment from patients who used less medication than prescribed, for example because of a period of hospitalization.

Reinitiation was defined as the dispensing of the same or a different class of antihypertensive drug within one year following the discontinuation. The proportion of patients reinitiating antihypertensives was calculated by dividing the number of patients who were dispensed beyond the end of the allowable maximum gap (> 180 days) by the number of patients who discontinued treatment. Patients were censored at the end of follow-up.

### Potential predictors of non-adherence, non-persistence and reinitiation

The variables considered to be potential predictors of non-adherence, non-persistence and reinitiation included sociodemographic factors and drug-related factors that could be measured using a pharmacy database. The potential predictors of non-adherence and non-persistence were assessed at initiation of the antihypertensive drug, while predictors of reinitiation were assessed within a 120 day period before the discontinuation date. The discontinuation date was defined as the theoretical end date of the last prescription. For patients using more than one class of antihypertensive drugs, the last theoretical end date was defined as the discontinuation date, regardless of whether an antihypertensive drug that was used simultaneously was discontinued earlier.

The sociodemographic factors were age, gender and socioeconomic status (SES). SES was obtained from neighbourhood status scores on four-digit postcode level based on evidence from the Netherlands Institute for Social Research.[16] A status score is an indicator comparing the social status of districts in the Netherlands, which is derived from a number of characteristics of the neighbourhood population (education, income and labour market position).[17] SES was classified into two groups, high or low, based on whether the score was above or below the median status score.

Drug-related factors included type of prevention, type of initial antihypertensive regimen, type of initial antihypertensive drug class, polypharmacy, drug proxies for conditions that may affect persistence and adherence, and type of prescriber. Type of prevention was defined as primary or secondary based on the prescription of a proxy indicating a previous cardiovascular event.[18] Patients who were dispensed at least two prescriptions of a platelet aggregation inhibitor, organic nitrate and/or a vitamin K antagonist in the year before the index date were categorized as secondary prevention patients. All others were considered primary prevention patients. The type of initial antihypertensive regimen was defined as monotherapy, free combination or fixed-dose combinations. Free combination was defined as treatment with two drug classes dispensed within seven days of the index date, while fixed-dose combination was defined as treatment with a drug containing a fixed dose of multiple substances. Polypharmacy was defined as chronic concurrent prescriptions for at least five drugs, excluding prescriptions with ATC codes D, V, Y, Z and prescriptions without an ATC code.[19] Chronic concurrent use was defined as dispensed for at least 90 days or at least two prescriptions within four months before the index date at pharmacological subgroup level (i.e. third level of ATC code). Drug proxies for conditions that may affect adherence and persistence were derived from specific drugs dispensed in the year before initiation (see list of drugs and ATC codes in S1 Table). Type of initial prescriber was classified as general practitioner or specialist. Duration of persistence for the reinitiation model was defined as the duration between the index date and the discontinuation date.

### Data analysis

Patient characteristics and outcome measures were reported using descriptive statistics. Chi-square tests were used to assess univariate associations of dichotomous or nominal variables with the binary outcomes. The potential predictors found to be associated with the outcome at a significance level of p < .25 in the univariate analysis (S2–S4 Tables) were included in the initial multivariate models. Since there was little missing data, we conducted complete-case analyses. Odds ratios (ORs) and hazard ratios (HRs) with a 95% confidence interval were obtained using logistic regression and Cox regression analyses respectively, with manual backward elimination. A sensitivity analysis was carried out for two periods of initiation of an antihypertensive drug. These periods were defined as before (1995–2007) or after (2008–2014) the implementation of an updated Dutch CVRM guideline and new disease management programmes which paid more attention to adherence monitoring. All statistical analyses were carried out using SPSS software (version 25.0; IBM, Armonk, NY, USA).

## Results

In total, we identified 6,669 diabetes patients in the IADB.nl pharmacy database who initiated an antihypertensive drug between 1995 and 2015 (Table 1). Among these 6,669 patients, non-adherence rates in persistent patients decreased from 11.0% in the first year to 8.5% and 7.7% in the second and third years, respectively. Non-persistence rates decreased from 18.0% in the first year to 3.7% and 2.9% in the second and third years, respectively. Of the 1,201 patients who discontinued the antihypertensive drug in the first year after initiation, 22.0% reinitiated treatment within one year following discontinuation. After the introduction of the new CVRM guideline and disease management programmes in 2007, non-adherence rates in persistent patients and non-persistence rates in the first year after initiation decreased from 11.8% to 9.6% (p-value: 0.012) and from 19.0% to 16.3% (p-value: 0.006), respectively. The numbers of non-adherent and non-persistent patients and those who reinitiated antihypertensive drugs over three years are presented in Fig 1.

**Table 1:** Characteristics of patients initiating an antihypertensive drug (N = 6,669).

Characteristic	Number	%
**Gender**		
Male	3,676	55.1
Female	2,993	44.9
**Age, mean [SD], years**	63.2 [11.3]	
**Age group, years**		
40–49	848	12.7
50–59	1,741	26.1
60–69	2,025	30.4
70–79	1,535	23.0
≥ 80	520	7.8
**Socioeconomic status**		
High	3,248	49.5
Low	3,310	50.5
Missing	111	
**Prevention type**		
Primary prevention	6,084	91.2
Secondary prevention	585	8.8
**Type of initial antihypertensive regimen**		
Monotherapy	6,587	98.8
Free combination	62	0.9
Fixed-dose combination	20	0.3
**Type of initial antihypertensive class**		
Diuretics	1,000	15.0
Beta-blocking agents	1,288	19.3
Calcium channel blockers	298	4.5
Agents acting on renin-angiotensin system	4,083	61.2
**Polypharmacy**		
Yes	755	11.3
No	5,914	88.7
**Type of initial prescriber**		
General practitioner	4,855	72.8
Specialist	198	3.0
Unknown	1,616	24.2
**Drug dispensed before antihypertensive initiation**		
Cardiovascular comorbidity	815	43.9
Psychiatric disorder	753	40.6
COPD	257	13.9
Other serious morbidity	29	1.6

Predictors of non-adherence were secondary prevention (OR: 1.45; 95% CI: 1.10–1.93) and the prescription of diuretics as initial drug class (OR: 1.37; 95% CI: 1.08–1.74) (Table 2). The goodness-of-fit p-value of the model was .963, with an R-squared value of 30%. The analysis of the different time periods, that is before (1995–2007) and after (2008–2014) the introduction of the new CVRM guideline, generally showed similar point estimates but not all estimates remained significant in both periods (S5 Table). Predictors of non-persistence were female gender (HR: 1.18; 95% CI: 1.05–1.32), older age (HR: 1.33; 95% CI: 1.08–1.63) and the prescription of diuretics, beta-blocking agents or calcium channel blockers as initial drug class (Table 3). The goodness-of-fit p-value of the model was .368, with an R-squared value of 12.4%. Again, the analysis of the different time periods generally showed similar point estimates for both periods but not all remained significant in both periods (S5 Table). The characteristics of patients at 120 days before discontinuation are shown in S6 Table. Longer duration of persistence (> 90 days) was a significant predictor of reinitiation (Table 4). The goodness-of-fit p-value of the reinitiation model was .342, with an R-squared value of 29.7%.

**Table 2:** Predictors of non-adherence to antihypertensive drugs in persistent patients in the first year after initiation.

Predictors^a^	Odds Ratios^b^ (95% CI)
**Prevention type**	
Primary prevention	Reference
Secondary prevention	1.45 (1.10–1.93)
**Type of initial antihypertensive class**	
Diuretics	1.37 (1.08–1.74)
Beta-blocking agents	1.19 (0.94–1.49)
Calcium channel blockers	1.24 (0.80–1.91)
Agents acting on renin-angiotensin system	Reference

^a^ goodness-of-fit p-value: .963; R-squared: 30%

^b^ final multivariate model

**Table 3:** Predictors of non-persistence to antihypertensive drugs in first year after initiation.

Predictors^a^	Hazard Ratios^b^ (95% CI)
**Gender**	
Male	Reference
Female	1.18 (1.05–1.32)
**Age group, years**	
40–49	1.09 (0.90–1.33)
50–59	1.02 (0.88–1.20)
60–69	Reference
70–79	1.11 (0.95–1.30)
≥ 80	1.33 (1.08–1.63)
**Type of initial antihypertensive class**	
Diuretics	1.60 (1.37–1.86)
Beta-blocking agents	1.16 (1.01–1.35)
Calcium channel blockers	2.09 (1.69–2.59)
Agents acting on renin-angiotensin system	Reference

^a^ goodness-of-fit p-value: .368; R-squared: 12.4%

^b^ final multivariate model

**Table 4:** Predictors of reinitiation of antihypertensive drug among non-persistent patients (N = 1,201).

Predictors^a^	Odds Ratios^b^ (95% CI)
**Duration of persistence, days**	
< 90	Reference
91–180	3.13 (1.94–5.05)
181–270	3.68 (2.12–6.39)
> 270	15.68 (10.80–22.79)

^a^ goodness-of-fit p-value: .342; R-squared: 29.7%

^b^ final multivariate model

## Discussion

Among patients on oral diabetes drugs who initiated an antihypertensive drug, both non-adherence rates in persistent patients and non-persistence rates decreased over the next three years. Non-adherence and non-persistence rates for these drugs were somewhat lower in the more recent time period. Among those who discontinued in the first year after initiation, more than one fifth reinitiated treatment within one year. Predictors of non-adherence included several drug-related factors, while predictors of non-persistence included sociodemographic and drug-related factors. Longer duration of persistence before discontinuation was a predictor of reinitiation.

Results from previous studies indicated that the first year of therapy has the highest risk of non-adherence to and non-persistence with antihypertensive drugs among patients with or without diabetes.[4,5] Our study showed that this is still the case when disentangling non-adherence from non-persistence by looking at non-adherence in persistent patients. The observed rates of non-adherence to and non-persistence with antihypertensive drugs were, however, lower than reported in other cohort studies among diabetes patients in Sweden or the US, for example.[20,21] In the Netherlands, lower non-adherence and non-persistence rates can be expected, since patients are obliged to have health insurance, which covers most of the costs for prescribed antihypertensive drugs. A previous literature review has reported that the reduction of drug expenses through better insurance coverage can improve drug adherence.[22] Another reason for the low rates of non-adherence and non-persistence in the Netherlands might be the organization of diabetes care, supported by electronic health systems in pharmacies and general practices, which helps to monitor chronic drug use.[23] This may be supported by our finding that the rates were lower after the implementation of new treatment guidelines and related disease management programmes. The Dutch national multidisciplinary guideline for cardiovascular risk management published in 2011 emphasized the importance of monitoring patient adherence to treatment.[24] Furthermore, the relatively low non-adherence rates in our study can in part be explained by our exclusion of non-persistent patients from the adherence calculation, which was similar to what we found in our previous study assessing non-adherence to statins.[23] The non-persistence rates in our study are in part driven by using a > 180 day gap definition for all antihypertensive drugs, which takes multiple drug use into account. A gap > 180 days is a relatively long period compared to that used in some of the other studies assessing non-persistence to antihypertensive drugs, either as multiple drugs or individual drugs, among diabetes[21] or non-diabetes patients.[25,26] Shorter gap lengths lead to higher discontinuation rates as they also include non-adherent patients, that is, patients who only used some, or needed less, of the drug than prescribed in the study period. Furthermore, we defined non-persistence as the discontinuation of all antihypertensive drugs, thereby taking drug switches into account. Therefore, we are certain that patients completely discontinued all antihypertensive drugs and that we did not misclassify patients who discontinued the initial drug but started or continued another antihypertensive drug.

We did not find previous studies reporting on reinitiation rates of antihypertensives in a high-risk population such as patients with diabetes. The reinitiation rate observed in our study was higher than that reported among a general group of new users of antihypertensive drugs in the Netherlands.[6] The latter study found that those patients who also used diabetes drugs at discontinuation–around 7% of the study population–were more likely to reinitiate antihypertensive treatment. Nevertheless, our study showed that the reinitiation rate in patients with diabetes was still low, considering that most of these patients initiating antihypertensive drugs are in need of chronic treatment. Nevertheless, some of the patients who did not reinitiate treatment in our study may not have needed continuous use of antihypertensive drugs. It should be noted that some of the patients who discontinued their antihypertensive drug within 90 days after initiation used low-ceiling diuretics (12.9%), which may have been intended for short-term use.

Looking at persistent patients, we observed that those who initiated diuretics were more likely to become non-adherent than those initiating agents acting on the renin-angiotensin system. This is in line with previous studies assessing non-adherence in patients with or without diabetes.[25,27–29] The difference in non-adherence by drug class can in part be explained by differences in dosing frequency.[21] Furthermore, we found that secondary prevention patients were more likely to become non-adherent than primary prevention patients. This result is contrary to a previous meta-analysis which showed that secondary prevention patients are more likely to be adherent to antihypertensive drugs.[30] However, patients on oral diabetes drugs were not included in that study, which might explain the difference in the findings. In our study, patients on oral diabetes drugs in need of secondary prevention were more likely polypharmacy patients (23.0%) compared to those in the primary prevention group (9.9%). Polypharmacy is a known factor associated with lower adherence.[31]

In our study, female patients were more likely to become non-persistent, while older age was also associated with more non-persistence, which is in line with a previous study assessing non-persistence with antihypertensive drugs among patients previously diagnosed with diabetes.[32] This could be due to the experience of more adverse effects. Female patients have reported higher rates of adverse events than males when using antihypertensive drugs.[9] In older patients, changes in drug metabolism may lower tolerance and increase the risk of side effects.[33] Patients initiating diuretics, beta-blocking agents or calcium channel blockers were more likely to become non-persistent than those initiating agents acting on the renin-angiotensin system. The difference in non-persistence by drug class may partially be explained by differences in drug tolerability[34], dosing frequency[21] and patient perception of side effects.[21,34] It should be noted that the prescription of these drugs as initial treatment is likely to decrease, given current recommendations to start with ARBs or ACEI in patients with diabetes.

Finally, we observed that patients with longer duration of persistence (> 90 days) before discontinuation were more likely to reinitiate antihypertensive drug treatment. Patients who reinitiated antihypertensive drug treatment were more likely to establish good drug-taking behaviour before discontinuation.[6] Overall, some factors were not associated with any aspect of drug-taking behaviour, in contrast to previous studies. In particular, socioeconomic status was not a predictor of non-adherence, non-persistence or reinitiation. This can be explained by the health insurance coverage of prescribed antihypertensive drugs in the Netherlands.

One strength of this study is the distinction we made between the dynamic process of non-adherence, non-persistence and reinitiation. This allowed us to identify specific predictors of these separate processes of drug-taking behaviour, which can be used to develop a tailored intervention. We also used adequate measures to assess non-adherence to any antihypertensive drug and non-persistence to all antihypertensive drugs, which considered multiple drugs used and drug switches.[15] Thus, we did not overestimate non-adherence or non-persistence when patients switched to another class of antihypertensive drugs. Furthermore, our use of a pharmacy database with good ascertainment of drug coverage is a strong point. This database has comprehensive information on all drugs dispensed in the population covered, since patients in the Netherlands usually collect all their medication from one pharmacy, where they are also registered. In addition, the advantage of using a pharmacy database is its provision of objective measurement, which is not biased by selection of patients. Information bias was also not likely to occur because this study indirectly assessed the medication used in the study population.

Some limitations need to be mentioned. Assessment of non-adherence and non-persistence based on drug dispensing is likely to underestimate true rates, since patients may not take all the drugs they collect at the pharmacy. Moreover, we could not correct for patients who may have been treated in hospitals or nursing homes for longer periods, which can also lead to overestimations. However, we chose a relatively long gap of 180 days to clearly distinguish patients who discontinued treatment from patients who were taking or needed fewer drugs than prescribed. We defined patients with type 2 diabetes based on the dispensing of at least two prescriptions for oral diabetes drugs. Patients only receiving insulin were thereby excluded, which might have resulted in the elimination of some patients with advanced type 2 diabetes. It is possible that some patients had a diagnosis other than hypertension. However, a previous study showed that antihypertensive drugs are mostly used for those diagnosed with hypertension.[35] Only about 20% of these drugs are used for angina pectoris, myocardial infarction or heart failure, and less than 1% for other indications, such as oedema.[35]

In addition, we excluded patients based on their use of specific medication that may be indicative of other diseases. We also lacked information to identify the role of the prescriber in discontinuation and reinitiation. The overall prediction of our non-persistence model was relatively low, indicating that there are other predictors which are not identifiable using a pharmacy database. Our use of a proxy to define prevention status could also lead to some misclassification. The proxy used in our study showed a sensitivity of 85% and a specificity of 75% in identifying patients with a history of major CVD.[18] Although this is rather low from a diagnostic perspective, the use of drug proxies is still useful to identify patients who may be less adherent to their medication. A small number of patients (0.9%) switched to other antihypertensive drug classes that were not included in our study.

In addition, we only included patients over 40 years of age, which might underestimate the number of patients with type 2 diabetes in the Netherlands. However, it has been reported that the number of type 2 diabetes patients who are younger than 40 is less than 1%.[36] Thus, it is expected that including younger age would not significantly influence our results. No causal inferences can be made regarding the introduction of the new guidelines and disease management programmes when comparing time periods.

Our findings highlight the need for pharmacists and other healthcare workers to monitor patients closely during the first year after initiation. Interventions should be tailored to different subgroups, since the predictors of non-adherence and non-persistence are not the same. Reasons for non-adherence may include forgetfulness, lack of understanding the medication regimen, and problems with taking the drugs.[37,38]. Reasons for non-persistence may be due to other factors, such as low necessity or high concerns beliefs.[39] Based on our study, we suggest that pharmacists and other healthcare workers need to provide strategies to cope with forgetfulness, including reminder or habit-based strategies as well as providing additional information to improve adherence in secondary prevention patients and those initiating diuretics. In addition, they need to support older patients and those initiating beta-blocking agents or calcium channel blockers in the persistent use of their drugs by reducing any high level concerns or low level necessity beliefs using motivational interviewing. In particular, more attention needs to be paid to patients who discontinue treatment after > 90 days without switching to another antihypertensive. When this concerns antihypertensives other than diuretics, patients are likely to be in need of chronic treatment. It is recommended that future studies assess the effect of individual antihypertensive drugs and their dosages in addition to class level effects on drug-taking behaviour. Moreover, it may be of value to study the effect of other drugs used by patients with diabetes on their antihypertensive drug-taking behaviour.

## Conclusions

Adherence to and persistence with antihypertensive drugs in patients on oral diabetes drugs has improved over time but remains suboptimal. The first year after initiation is the most crucial with regard to non-adherence and non-persistence. Since only one fifth of all patients reinitiated treatment within one year after discontinuation, more attention should be paid to these patients during this period. Interventions should be tailored, since the predictors of non-adherence, non-persistence and reinitiation are not the same.

## Supporting information

S1 Fig Definition of adherent, non-persistent and reinitiation. (TIF)

S1 Table List of drugs with ATC code. (DOCX)

S2 Table Univariate associations with non-adherence to antihypertensive drugs in persistent patients (N = 5,468). (DOCX)

S3 Table Univariate associations with non-persistence to antihypertensive drug (N = 6,669). (DOCX)

S4 Table Univariate associations of characteristics at discontinuation with reinitiation of antihypertensive drug (N = 1,201). (DOCX)

S5 Table Sensitivity analysis of predictors of non-adherence in persistent patients and non-persistence based on period of initiation of antihypertensive drug. (DOCX)

S6 Table Characteristics of patients at 120 days before discontinuation* (N = 1,201). (DOCX)

## References

[1] American Diabetes Association. 10. Cardiovascular Disease and Risk Management: Standards of Medical Care in Diabetes—2019. Diabetes Care. 2019;42: S103–S123. 10.2337/dc19-S010 30559236

[2] Stalman WAB, Scheltens T, Burgers JS, Hukkelhoven CWPM, Smorenburg SM, Banga JD et al Dutch guideline cardiovascular risk management 2006 [Internet]. [cited 3 Jul 2019]. Available: http://www.nhg.org

[3] Ledur PS, Leiria LF, Severo MD, Silveira DT, Massierer D, Becker AD, et al Perception of uncontrolled blood pressure and non-adherence to anti-hypertensive agents in diabetic hypertensive patients. J Am Soc Hypertens. Elsevier; 2013;7: 477–483. 10.1016/J.JASH.2013.07.006 23969287

[4] Burke TA, Sturkenboom MC, Lu S, Wentworth CE, Lin Y, Rhoads GG. Discontinuation of antihypertensive drugs among newly diagnosed hypertensive patients in UK general practice. J Hypertens. 2006;24: 1193–1200. 10.1097/01.hjh.0000226211.95936.f5 16685222

[5] Sung SK, Lee SG, Lee KS, Kim DS, Kim KH, Kim KY. First-year treatment adherence among outpatients initiating antihypertensive medication in Korea: Results of a retrospective claims review. Clin Ther. 2009;31: 1309–1320. 10.1016/j.clinthera.2009.06.011 19695396

[6] Van Wijk BLG, Avorn J, Solomon DH, Klungel OH, Heerdink ER, De Boer A, et al Rates and determinants of reinitiating antihypertensive therapy after prolonged stoppage: A population-based study. J Hypertens. 2007;25: 689–697. 10.1097/HJH.0b013e3280148a58 17278986

[7] Wijk Van BLG, Klungel OH, Heerdink ER, de Boer A. Rate and determinants of 10-year persistence with antihypertensive drugs. J Hypertens. 2005;23: 2101–2107. 10.1097/01.hjh.0000187261.40190.2e 16208154

[8] Bourgault C, Sénécal M, Brisson M, Marentette M, Grégoire J-P. Persistence and discontinuation patterns of antihypertensive therapy among newly treated patients: a population-based study. J Hum Hypertens. 2005;19: 607–613. 10.1038/sj.jhh.1001873 15920457

[9] Degli Esposti E, Sturani A, Falasca P, Novi M, Baio G, Buda S, et al Long-term persistence with antihypertensive drugs in new patients. J Hum Hypertens. 2002;16: 439–444. 10.1038/sj.jhh.1001418 12037702

[10] Vrijens B, De Geest S, Hughes DA, Przemyslaw K, Demonceau J, Ruppar T, et al A new taxonomy for describing and defining adherence to medications. Br J Clin Pharmacol. 2012;73: 691–705. 10.1111/j.1365-2125.2012.04167.x 22486599 PMC3403197

[11] Halpern MT, Khan ZM, Schmier JK, Burnier M, Caro JJ, Cramer J, et al Recommendations for evaluating compliance and persistence with hypertension therapy using retrospective data. Hypertension. 2006;47: 1039–1048. 10.1161/01.HYP.0000222373.59104.3d 16651464

[12] De Geest S, Zullig LL, Dunbar-Jacob J, Helmy R, Hughes DA, Wilson IB, et al ESPACOMP medication adherence reporting guideline (EMERGE). Ann Intern Med. 2018;169: 30–35. 10.7326/M18-0543 29946690 PMC7643841

[13] Sediq R, van der Schans J, Dotinga A, Alingh R, Wilffert B, Bos JH, et al Concordance assessment of self-reported medication use in the Netherlands three-generation Lifelines Cohort Study with the pharmacy database IADB.nl: The PharmLines Initiative. Clin Epidemiol. Dove Press; 2018;10: 981–989. 10.2147/CLEP.S163037 30147377 PMC6101003

[14] Overheid. N. Wet medisch wetenschappelijk onderzoek met mensen. [Internet]. [cited 1 Aug 2019]. Available: http://wetten.overheid.nl/BWBR0009408/2013-09-27/1

[15] Alfian SD, Pradipta IS, Hak E, Denig P. A systematic review finds inconsistency in the measures used to estimate adherence and persistence to multiple cardiometabolic medications. J Clin Epidemiol. Pergamon; 2019;108: 44–53. 10.1016/j.jclinepi.2018.12.003 30537541

[16] Frans K, Jeroen B, Veldheer V. Neighbourhood status development in the Netherlands 1998–2010—SCP English [Internet]. 2012 [cited 27 Sep 2016]. Available: https://www.scp.nl/

[17] Jansen T, Zwaanswijk M, Hek K, de Bakker D. To what extent does sociodemographic composition of the neighbourhood explain regional differences in demand of primary out-of-hours care: a multilevel study. BMC Fam Pract. 2015;16: 54 10.1186/s12875-015-0275-0 25943593 PMC4424822

[18] Pouwels KB, Voorham J, Hak E, Denig P. Identification of major cardiovascular events in patients with diabetes using primary care data. BMC Health Serv Res. 2016;16: 110 10.1186/s12913-016-1361-2 27038959 PMC4818875

[19] Nederlands Huisarten Genootschap. Multidisciplinaire Richtlijn Polyfarmacie bij ouderen 2012 [Internet]. 2012 [cited 1 Apr 2018]. Available: https://www.nhg.org/sites/default/files/content/nhg_org/uploads/polyfarmacie_bij_ouderen.pdf

[20] Hedna K, Hakkarainen KM, Gyllensten H, Jönsson AK, Sundell KA, Petzold M, et al Adherence to antihypertensive therapy and elevated blood pressure: should we consider the use of multiple medications? PLoS One. 2015;10: 1–14. 10.1371/journal.pone.0137451 26359861 PMC4567373

[21] Friedman O, McAlister FA, Yun L, Campbell NRC, Tu K. Antihypertensive drug persistence and compliance among newly treated elderly hypertensives in Ontario. Am J Med. 2010;123: 173–181. 10.1016/j.amjmed.2009.08.008 20103027

[22] Lee JL, Maciejewski ML, Raju SS, Shrank WH, Choudhry NK. Value-Based Insurance Design: Quality Improvement But No Cost Savings. Health Aff. 2013;32: 1251–1257. 10.1377/hlthaff.2012.0902 23836741

[23] Alfian SD, Worawutputtapong P, Schuiling-Veninga CCM, van der Schans J, Bos JHJ, Hak E, et al Pharmacy-based predictors of non-persistence with and non-adherence to statin treatment among patients on oral diabetes medication in the Netherlands. Curr Med Res Opin. 2018;34: 1013–1019. 10.1080/03007995.2017.1417242 29292657

[24] Wiersma T, Smulders YM, Stehouwer CD, Konings KT, Lanphen J. Summary of the multidisciplinary guideline on cardiovascular risk management (revision 2011). Ned Tijdschr Geneeskd. 2012;156: A5104 22951134

[25] Baggarly SA, Kemp RJ, Wang X, Magoun AD. Factors associated with medication adherence and persistence of treatment for hypertension in a Medicaid population. Res Soc Adm Pharm. Elsevier Inc; 2014;10: 99–112. 10.1016/j.sapharm.2014.02.002 24731547

[26] Erkens JA, Panneman MMJ, Klungel OH, Boom G van den, Prescott MF, Herings RMC. Differences in antihypertensive drug persistence associated with drug class and gender: a PHARMO study. Pharmacoepidemiol Drug Saf. John Wiley & Sons, Ltd.; 2005;14: 795–803. 10.1002/pds.1156 16178043

[27] Kronish IM, Woodward M, Sergie Z, Ogedegbe G, Falzon L, Mann DM. Meta-analysis: impact of drug class on adherence to antihypertensives. Circulation. 2011;123: 1611–1621. 10.1161/CIRCULATIONAHA.110.983874 21464050 PMC3084582

[28] Siegel D, Lopez J, Meier J. Antihypertensive medication adherence in the Department of Veterans Affairs. Am J Med. 2007;120: 26–32. 10.1016/j.amjmed.2006.06.028 17208076

[29] Elliott WJ, Plauschinat CA, Skrepnek GH, Gause D. Persistence, adherence, and risk of discontinuation associated with commonly prescribed antihypertensive drug monotherapies. J Am Board Fam Med. American Board of Family Medicine; 2007;20: 72–80. 10.3122/jabfm.2007.01.060094 17204738

[30] Naderi SH, Bestwick JP, Wald DS. Adherence to drugs that prevent cardiovascular disease: Meta-analysis on 376,162 patients. Am J Med. Elsevier Inc.; 2012;125: 882–887. 10.1016/j.amjmed.2011.12.013 22748400

[31] Cramer JA. A systematic review of adherence with medications for diabetes. Diabetes Care. 2004;27: 1218–1224. 10.2337/diacare.27.5.1218 15111553

[32] Tu K, Anderson LN, Butt DA, Quan H, Hemmelgarn BR, Campbell NR, et al Antihypertensive drug prescribing and persistence among new elderly users: Implications for persistence improvement interventions. Can J Cardiol. Canadian Cardiovascular Society; 2014;30: 647–652. 10.1016/j.cjca.2014.03.017 24882536

[33] Klotz U. Pharmacokinetics and drug metabolism in the elderly. Drug Metab Rev. 2009;41: 67–76. 10.1080/03602530902722679 19514965

[34] Hasford J, Schröder-bernhardi D, Rottenkolber M, Kostev K, Dietlein G. Persistence with antihypertensive treatments: results of a 3-year follow-up cohort study. Eur J Clin Pharmacol. 2007;63: 1055–1061. 10.1007/s00228-007-0340-2 17701032

[35] Poluzzi E, Strahinja P, Vargiu A, Chiabrando G, Silvani MC, Motola D, et al Initial treatment of hypertension and adherence to therapy in general practice in Italy. Eur J Clin Pharmacol. Springer-Verlag; 2005;61: 603–609. 10.1007/s00228-005-0957-y 16082539

[36] Centraal Bureau voor de Statistiek. More and more people with diabetes [Internet]. 2014 [cited 21 May 2019]. Available: https://www.cbs.nl/en-gb/news/2014/46/more-and-more-people-with-diabetes

[37] Wroe AL. Intentional and unintentional nonadherence: a study of decision making. J Behav Med. Kluwer Academic Publishers-Plenum Publishers; 2002;25: 355–372. 10.1023/a:1015866415552 12136497

[38] Clifford S, Barber N, Horne R. Understanding different beliefs held by adherers, unintentional nonadherers, and intentional nonadherers: Application of the Necessity-Concerns Framework. J Psychosom Res. 2008;64: 41–46. 10.1016/j.jpsychores.2007.05.004 18157998

[39] McHorney C. Individual patients hold different beliefs to prescription medications to which they persist vs nonpersist and persist vs nonfulfill. Patient Prefer Adherence. 2010; 187 10.2147/PPA.S10603 PMC2915553 20694180

