# Supplementary figures and images for: Pharmacy-based predictors of non-adherence, non-persistence and reinitiation of antihypertensive drugs among patients on oral diabetes drugs in the Netherlands
Source: PLoS One. 2019 Nov 15;14(11):e0225390. doi: 10.1371/journal.pone.0225390 (PMC6857926; doi:10.1371/journal.pone.0225390)

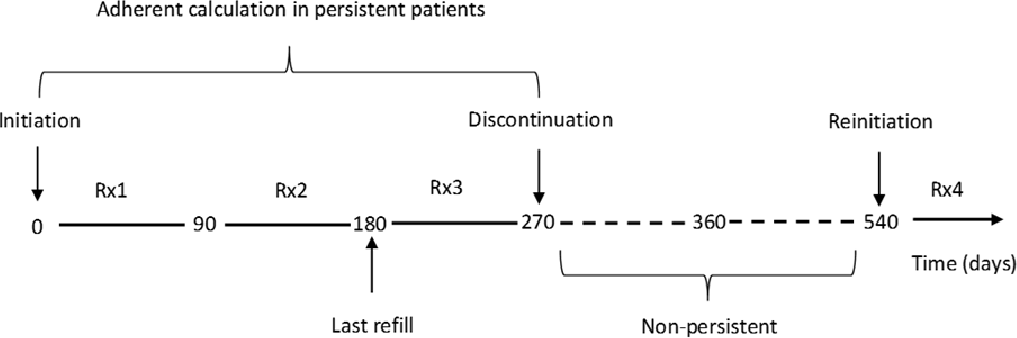

Supplement: S1 Fig — (TIF) [file pone.0225390.s001.tif]
