# Supplementary material for: Pharmacy-based predictors of non-adherence, non-persistence and reinitiation of antihypertensive drugs among patients on oral diabetes drugs in the Netherlands
Source: PLoS One. 2019 Nov 15;14(11):e0225390. doi: 10.1371/journal.pone.0225390 (PMC6857926; doi:10.1371/journal.pone.0225390)
Supplement: S1 Table — (DOCX) [file pone.0225390.s002.docx]

**Table S1. List of drugs with ATC code.**

| **No** | **Drugs** | **ATC code** |
| --- | --- | --- |
| 1 | Non-insulin blood glucose-lowering drug | A10B |
| 2 | Diuretics | C03 |
| 3 | Beta blocking agents | C07 |
| 4 | Calcium channel blockers | C08 |
| 5 | Agents acting on the renin-angiotensin system | C09 |
| 6 | Propanolol | C07AA05 |
| 7 | High-ceiling diuretics | C03C |
| 8 | Platelet aggregation inhibitors | B01AC |
| 9 | Organic nitrates | C01DA |
| 10 | Vitamin K antagonists | B01AA |
| 11 | FDC enalapril and diuretics | C09BA02 |
| 12 | Antithrombotic agents | B01 excluding B01AC and B01AA |
| 13 | Cardiac therapy | C01 excluding C01DA |
| 14 | Lipid modifying agents | C10 |
| 15 | Antipsychotics | N05A |
| 16 | Anxiolytics | N05B |
| 17 | Hypnotics and sedatives | N05C |
| 18 | Antidepressants | N06A |
| 19 | Glucocorticoids | R03BA |
| 20 | Anticholinergics | R03BB |
| 21 | Selective beta-2-adrenoreceptor agonists | R03AC |
| 22 | Xanthines | R03DA |
| 23 | Antineoplastic agents | L01 |
| 24 | Immunostimulants | L03 |
| 25 | Immunosuppressants | L04 |

Abbreviations: ATC = Anatomical Therapeutic Chemical; FDC = Fixed-dose combination
