# Supplementary material for: Pharmacy-based predictors of non-adherence, non-persistence and reinitiation of antihypertensive drugs among patients on oral diabetes drugs in the Netherlands
Source: PLoS One. 2019 Nov 15;14(11):e0225390. doi: 10.1371/journal.pone.0225390 (PMC6857926; doi:10.1371/journal.pone.0225390)
Supplement: S2 Table — (DOCX) [file pone.0225390.s003.docx]

**Table S2. Univariate associations with non-adherence to antihypertensive drugs in persistent patients (N= 5,468).**

| **Potential predictors** | **Adherence**  **N (%)** | **Non-adherence**  **N (%)** | ***P* value** |
| --- | --- | --- | --- |
| **Gender (N)** |  |  | 0.270 |
| Male (3,068) | 2,717 (55.8) | 351 (58.2) |  |
| Female (2,400) | 2,148 (44.2) | 252 (41.8) |  |
| **Age group, years (N)** |  |  |  |
| 40-49 (695) | 611 (12.6) | 84 (13.9) | 0.791 |
| 50-59 (1,453) | 1,287 (26.5) | 166 (27.5) |  |
| 60-69 (1,681) | 1,502 (30.9) | 179 (29.7) |  |
| 70-79 (1,243) | 1,109 (22.8) | 134 (22.2) |  |
| ≥ 80 (396) | 356 (7.3) | 40 (6.6) |  |
| **Socioeconomic status (N)** |  |  | 0.699 |
| High (2,683) | 2,391 (50.0) | 292 (49.2) |  |
| Low (2,693) | 2,391 (50.0) | 302 (50.8) |  |
| Missing (92) |  |  |  |
| **Prevention type (N)** |  |  |  |
| Primary prevention (4,986) | 4,457 (91.6) | 529 (87.7) | 0.002* |
| Secondary prevention (482) | 408 (8.4) | 74 (12.3) |  |
| **Type of initial antihypertensive regimen (N)** | |  | 0.447 |
| Monotherapy (5,395) | 4,803 (98.7) | 592 (98.2) |  |
| Free combination (55) | 46 (0.9) | 9 (1.5) |  |
| Fixed-dose combination (18) | 16 (0.3) | 2 (0.3) |  |
| **Type of initial antihypertensive class (N)** | |  | 0.014* |
| Diuretics (760) | 660 (13.6) | 100 (16.6) |  |
| Beta-blocking agents (1,000) | 874 (18.0) | 126 (20.9) |  |
| Calcium channel blockers (199) | 173 (3.6) | 26 (4.3) |  |
| Agents acting on renin-angiotensin system (3,509) | 3,158 (64.9) | 351 (58.2) |  |
| **Polypharmacy (N)** |  |  | 0.128* |
| Yes (607) | 529 (10.9) | 78 (12.9) |  |
| No (4,861) | 4,336 (89.1) | 525 (87.1) |  |
| **Type of initial prescriber (N)** |  |  | 0.764 |
| General practitioner (3,994) | 3,561 (73.2) | 433 (71.8) |  |
| Specialist (161) | 142 (2.9) | 19 (3.2) |  |
| Unknown (1,313) | 1,162 (23.9) | 151 (25.0) |  |
| **Drug dispensed before antihypertensive initiation (N)** | |  |  |
| Cardiovascular disease (690) | 626 (12.9) | 64 (10.6) | 0.116* |
| Psychiatric disorder (601) | 535 (11.0) | 66 (10.9) | 0.969 |
| COPD (206) | 180 (3.7) | 26 (4.3) | 0.457 |
| Serious morbidity (23) | 18 (0.4) | 5 (0.8) | 0.100* |

Abbreviations: COPD: chronic obstructive pulmonary disease; * included in initial multivariate model
