# Supplementary material for: Pharmacy-based predictors of non-adherence, non-persistence and reinitiation of antihypertensive drugs among patients on oral diabetes drugs in the Netherlands
Source: PLoS One. 2019 Nov 15;14(11):e0225390. doi: 10.1371/journal.pone.0225390 (PMC6857926; doi:10.1371/journal.pone.0225390)
Supplement: S3 Table — (DOCX) [file pone.0225390.s004.docx]

**Table S3. Univariate associations with non-persistence to antihypertensive drug (N=6,669).**

| **Potential predictors** | **Persistence**  **N (%)** | **Non-persistence**  **N (%)** | ***P* value** | |  |
| --- | --- | --- | --- | --- | --- |
| **Gender (N)** |  |  |  | |  |
| Male (3,676) | 3,068 (56.1) | 608 (50.6) | 0.001* | |  |
| Female (2,993) | 2,400 (43.9) | 593 (49.4) |  | |  |
| **Age group, years (N)** |  |  |  | |  |
| 40-49 (848) | 695 (12.7) | 153 (12.7) | 0.002* | |  |
| 50-59 (1,741) | 1,453 (26.6) | 288 (24.0) |  | |  |
| 60-69 (2,025) | 1,681 (30.7) | 344 (28.6) |  | |  |
| 70-79 (1,535) | 1,243 (22.7) | 292 (24.3) |  | |  |
| ≥ 80 (520) | 396 (7.2) | 124 (10.3) |  | |  |
| **Socioeconomic status (N)** |  |  | 0.201* | |  |
| High (3,248) | 2,682 (49.9) | 566 (47.8) |  | |  |
| Low (3,310) | 2,693 (50.1) | 617 (52.2) |  | |  |
| Missing (111) |  |  |  | |  |
| **Prevention type (N)** |  |  |  | |  |
| Primary prevention (6,084) | 4,986 (91.2) | 1,098 (91.4) | 0.791 | |  |
| Secondary prevention (585) | 482 (8.8) | 103 (8.6) |  | |  |
| **Type of initial antihypertensive regimen** |  |  | 0.247* | |  |
| Monotherapy (6,587) | 5,395 (98.7) | 1,192 (99.3) |  | |  |
| Free combination (62) | 55 (1.0) | 7 (0.6) |  | |  |
| Fixed-dose combination (20) | 18 (0.3) | 2 (0.2) |  | |  |
| **Type of initial antihypertensive class (N)** |  |  | | <0.001* | |
| Diuretics (1,000) | 760 (13.9) | 240 (20.0) |  | |  |
| Beta-blocking agents (1,288) | 1,000 (18.3) | 288 (24.0) |  | |  |
| Calcium channel blockers (298) | 199 (3.6) | 99 (8.2) |  | |  |
| Agents acting on renin-angiotensin system (4,083) | 3,509 (64.2) | 574 (47.8) |  | |  |
| **Polypharmacy (N)** |  |  | 0.226* | |  |
| Yes (755) | 607 (11.1) | 148 (12.3) |  | |  |
| No (5,914) | 4,861 (88.9) | 1,053 (87.7) |  | |  |
| **Type of initial prescriber (N)** |  |  | 0.634 | |  |
| General practitioner (4,855) | 3,994 (73.0) | 861 (71.7) |  | |  |
| Specialist (198) | 161 (2.9) | 37 (3.1) |  | |  |
| Unknown (1,616) | 1,313 (24.0) | 303 (25.2) |  | |  |
| **Drug dispensed before antihypertensive initiation (N)** | |  |  | |  |
| Cardiovascular disease (815) | 690 (12.6) | 125 (10.4) | 0.034* | |  |
| Psychiatric disorder (753) | 601 (11.0) | 152 (12.7) | 0.099* | |  |
| COPD (257) | 206 (3.8) | 51 (4.2) | 0.435 | |  |
| Serious morbidity (29) | 23 (0.4) | 6 (0.5) | 0.707 | |  |

Abbreviations: COPD: chronic obstructive pulmonary disease; * included in initial multivariate model
