# Supplementary material for: Pharmacy-based predictors of non-adherence, non-persistence and reinitiation of antihypertensive drugs among patients on oral diabetes drugs in the Netherlands
Source: PLoS One. 2019 Nov 15;14(11):e0225390. doi: 10.1371/journal.pone.0225390 (PMC6857926; doi:10.1371/journal.pone.0225390)
Supplement: S4 Table — (DOCX) [file pone.0225390.s005.docx]

**Table S4. Univariate associations of characteristic at discontinuation with reinitiation to antihypertensive drug (N= 1,201).**

| **Potential predictors** | **Reinitiate**  **N (%)** | **No reinitiate**  **N (%)** | ***P* value** |
| --- | --- | --- | --- |
| **Gender (N)** |  |  | 0.420 |
| Male (608) | 142 (52.8) | 466 (50.0) |  |
| Female (593) | 127 (47.2) | 466 (50.0) |  |
| **Age group, years (N)** |  |  |  |
| 40-49 (144) | 32 (11.9) | 112 (12.0) | 0.999 |
| 50-59 (283) | 63 (23.4) | 220 (23.6) |  |
| 60-69 (339) | 77 (28.6) | 262 (28.1) |  |
| 70-79 (296) | 67 (24.9) | 229 (24.6) |  |
| ≥ 80 (139) | 30 (11.2) | 109 (11.7) |  |
| **Socioeconomic status (N)** |  |  | 0.391 |
| High (590) | 127 (47.6) | 463 (50.5) |  |
| Low (593) | 140 (52.4) | 453 (49.5) |  |
| Missing (18) |  |  |  |
| **Type of antihypertensive class (N)** | |  | 0.352 |
| Diuretics (240) | 55 (20.4) | 185 (19.8) |  |
| Beta-blocking agents (288) | 57 (21.2) | 231 (24.8) |  |
| Calcium channel blockers (99) | 18 (6.7) | 81 (8.7) |  |
| Agents acting on renin-angiotensin system (574) | 139 (51.7) | 435 (46.7) |  |
| **Polypharmacy (N)** |  |  | 0.272 |
| Yes (280) | 56 (20.8) | 224 (24.0 |  |
| No (921) | 213 (79.2) | 708 (76.0) |  |
| **Type of prescriber (N)** |  |  | 0.108* |
| General practitioner (861) | 185 (68.8) | 676 (72.5) |  |
| Specialist (37) | 5 (1.9) | 32 (3.4) |  |
| Unknown (303) | 79 (29.4) | 224 (24.0) |  |
| **Duration of persistence, days (N)** |  |  | <0.001* |
| < 90 (640) | 48 (17.8) | 592 (63.5) |  |
| 91-180 (168) | 34 (12.6) | 134 (14.4) |  |
| 181-270 (100) | 23 (8.6) | 77 (8.3) |  |
| >270 (293) | 164 (61.0) | 129 (13.8) |  |

Note: * included in initial multivariate model
