# Supplementary material for: Pharmacy-based predictors of non-adherence, non-persistence and reinitiation of antihypertensive drugs among patients on oral diabetes drugs in the Netherlands
Source: PLoS One. 2019 Nov 15;14(11):e0225390. doi: 10.1371/journal.pone.0225390 (PMC6857926; doi:10.1371/journal.pone.0225390)
Supplement: S5 Table — (DOCX) [file pone.0225390.s006.docx]

**Table S5. Sensitivity analysis of predictors of non-adherence in persistent patients and non-persistence based on period of initiation of antihypertensive drug.**

| **Predictors** | **Non-Adherence in persistent patients** | | **Non-persistence** | |
| --- | --- | --- | --- | --- |
|  | **Odds Ratio (OR) (95% CI)** | | **Hazard Ratio (HR) (95% CI)** | |
|  | **1995-2007 (N=3,453)** | **2008-2014 (N=2,015)** | **1995-2007 (N=4,262)** | **2008-2014**  **(N=2,407)** |
| **Rate in first year (%)** | 11.8 (n=409) | 9.6 (n=194) | 19.0 (n=809) | 16.3 (n=392) |
| **Gender** |  |  |  |  |
| Male | - | - | Reference | Reference |
| Female | - | - | 1.06 (0.92-1.22) | 1.39 (1.14-1.70) |
| **Age group, years** |  |  |  |  |
| 40-49 | - | - | 1.03 (0.81-1.32) | 1.19 (0.88-1.62) |
| 50-59 | - | - | 1.08 (0.89-1.31) | 0.93 (0.71-1.21) |
| 60-69 | - | - | Reference | Reference |
| 70-79 | - | - | 1.13 (0.93-1.36) | 1.02 (0.77-1.36) |
| ≥ 80 | - | - | 1.23 (0.96-1.57) | 1.49 (1.02-2.19) |
| **Prevention type** |  |  |  |  |
| Primary prevention | Reference | Reference | - | - |
| Secondary prevention | 1.36 (0.96-1.94) | 1.71 (1.05-2.78) | - | - |
| **Type of initial antihypertensive class** | |  |  |  |
| Diuretics | 1.68 (1.26-2.25) | 1.07 (0.69-1.64) | 1.82 (1.52-2.19) | 1.29 (0.99-1.70) |
| Beta-blocking agents | 1.03 (0.77-1.37) | 1.47 (0.99-2.19) | 1.11 (0.93-1.33) | 1.25 (0.97-1.61) |
| Calcium channel blockers | 1.15 (0.69-1.92) | 1.82 (0.79-4.16) | 1.87 (1.44-2.44) | 2.56 (1.77-3.69) |
| Agents acting on renin-angiotensin system | Reference | Reference | Reference | Reference |
