# Supplementary material for: Pharmacy-based predictors of non-adherence, non-persistence and reinitiation of antihypertensive drugs among patients on oral diabetes drugs in the Netherlands
Source: PLoS One. 2019 Nov 15;14(11):e0225390. doi: 10.1371/journal.pone.0225390 (PMC6857926; doi:10.1371/journal.pone.0225390)
Supplement: S6 Table — (DOCX) [file pone.0225390.s007.docx]

**Table S6. Characteristics of patients at 120 days before discontinuation* (N=1,201).**

| **Characteristic** | **Number** | **%** |
| --- | --- | --- |
|  | | |
| **Gender** |  |  |
| Male | 608 | 50.6 |
| Female | 593 | 49.4 |
| **Age, mean [SD], years** | 64.3 [12.0] |  |
| **Age group, years** |  |  |
| 40-49 | 144 | 12.0 |
| 50-59 | 283 | 23.6 |
| 60-69 | 339 | 28.2 |
| 70-79 | 296 | 24.6 |
| ≥ 80 | 139 | 11.6 |
| **Socioeconomic status** |  |  |
| High | 593 | 50.1 |
| Low | 590 | 49.9 |
| Missing | 18 |  |
| **Type of antihypertensive class** | | |
| Diuretics | 240 | 20.0 |
| Beta-blocking agents | 288 | 24.0 |
| Calcium channel blockers | 99 | 8.2 |
| Agents acting on renin-angiotensin system | 574 | 47.8 |
| **Polypharmacy** |  |  |
| Yes | 280 | 23.3 |
| No | 921 | 76.7 |
| **Type of prescriber** |  |  |
| General practitioner | 861 | 71.7 |
| Specialist | 37 | 3.1 |
| Unknown | 303 | 25.2 |
| **Duration of persistence, days** |  |  |
| < 90 | 640 | 53.3 |
| 91-180 | 168 | 14.0 |
| 181-270 | 100 | 8.3 |
| >270 | 293 | 24.4 |

Abbreviations: SD: standard deviation

Note: *the theoretical end date of the last prescription
